# Maternal Macronutrient Intake and Associated Risk for Gestational Diabetes Mellitus: Results from the BORN2020 Study

**DOI:** 10.3390/biomedicines13010057

**Published:** 2024-12-29

**Authors:** Antigoni Tranidou, Ioannis Tsakiridis, Emmanuela Magriplis, Aikaterini Apostolopoulou, Violeta Chroni, Eirini Tsekitsidi, Ioustini Kalaitzopoulou, Nikolaos Pazaras, Michail Chourdakis, Themistoklis Dagklis

**Affiliations:** 1 3rd Department of Obstetrics and Gynecology, School of Medicine, Faculty of Health Sciences, Aristotle University of Thessaloniki, 541 24 Thessaloniki, Greece; antigoni.tranidou@gmail.com (A.T.); igtsakir@auth.gr (I.T.); katapost@yahoo.gr (A.A.); nikospaz@hotmail.com (N.P.); 2 Laboratory of Hygiene, Social & Preventive Medicine and Medical Statistics, School of Medicine, Faculty of Health Sciences, Aristotle University of Thessaloniki, 541 24 Thessaloniki, Greece; violetac@auth.gr (V.C.); eirhnh-2@hotmail.com (E.T.); ioustinik@auth.gr (I.K.); mhourd@auth.gr (M.C.); 3 Department of Food Science and human Nutrition, Agricultural University of Athens, Iera Odos 75, 118 55 Athens, Greece; emagriplis@eatsmart.gr

**Keywords:** maternal, nutritional intake, pregnancy, gestational diabetes mellitus, GDM, macronutrients, dietary reference values, DRVs, European authority safety measures, EFSA

## Abstract

**Background/Objectives:** Limited evidence links maternal macronutrient intake to gestational diabetes mellitus (GDM) risk. Therefore, we evaluated these intakes both before and during pregnancy, comparing macronutrient data against the European Food and Safety Authorities’ (EFSA) Dietary Reference Values (DRVs). **Methods:** Data were prospectively collected from the Greek BORN2020 epidemiologic pregnant cohort, which included 797 pregnant women, of whom 14.7% were diagnosed with GDM. A multinomial logistic regression model assessed the association between macronutrient intake and GDM, adjusting for maternal, lifestyle, and pregnancy-related factors. **Results:** Women with GDM had higher maternal age (34.15 ± 4.48 vs. 32.1 ± 4.89 years), higher pre-pregnancy BMI (median 23.7 vs. 22.7 kg/m^2^), and were more likely to smoke during mid-gestation (17.95% vs. 8.82%). Pre-pregnancy energy intake exceeding EFSA recommendations was associated with increased GDM risk (aOR = 1.99, 95%CI: 1.37–2.86). During mid-gestation, higher dietary fiber intake (aOR = 1.05, 95%CI: 1.00–1.10), higher protein intake (aOR = 1.02, 95% CI: 1.00–1.04), and higher protein percentage of energy intake (aOR = 1.08, 95%CI: 1.01–1.17) were all significantly associated with increased GDM risk. Changes from pre-pregnancy to pregnancy showed significant increases in dietary fiber intake (aOR = 1.07, 95%CI: 1.04–1.10), protein (aOR = 1.00, 95%CI: 1.00–1.01), fat (aOR = 1.00, 95%CI: 1.00–1.01), vegetable protein (aOR = 1.01, 95%CI: 1.00–1.03), animal protein (aOR = 1.00, 95%CI: 1.00–1.01), and monounsaturated fatty acid (MUFA) intake (aOR = 1.01, 95%CI: 1.00–1.02), all of which were associated with increased GDM risk. **Conclusions:** Energy intake above upper levels set by EFSA, as well as increased protein, MUFA, and fiber intake, although beneficial in balanced intakes, may negatively affect gestation by increasing GDM likelihood when consumed beyond requirements.

## 1. Introduction

Appropriate macronutrient intake is crucial for both maternal health and fetal development, playing a key role in reducing gestational diabetes mellitus (GDM) risk and minimizing its associated complications [1]. A balanced macronutrient composition includes carbohydrates, which are vital for providing energy, maintaining stable maternal glucose levels, and supporting fetal brain development; proteins, which also contribute to the growth of fetal tissues, placenta formation, and maternal muscle maintenance [2]; and healthy fats, in particular omega-3 and omega-6 fatty acids, which are essential for fetal brain and eye development [3]. As pregnancy progresses, normal physiological changes lead to reduced insulin sensitivity and increased insulin resistance, characterized by hyperglycemia and hyperinsulinemia [4]. These changes are designed to prioritize glucose delivery to the fetus but may result in glucose intolerance if the maternal pancreas cannot produce sufficient insulin to compensate. These physiologic changes, in combination with improper nutritional intake, such as diets high in fat, may exacerbate glucose abnormalities and further aggravate this intolerance [5]. In addition, improper diets, such as those characterized by excessive fat intake, may link to GDM recurrence in future pregnancies [6].

While the type and proportion of macronutrients consumed during pregnancy may substantially influence GDM risk [7], recent data also underscore the importance of maternal nutritional status before conception [8]. Preconception nutrition lays the ground for optimal maternal metabolic health, influencing insulin sensitivity and glucose regulation, which, in turn, are associated with GDM risk. Results from the NHS Cohort Study indicated that increased intake of animal protein prior to conception may be associated with poor metabolic health, increasing the risk for GDM [9]. Maternal dietary patterns during pregnancy may be assessed indirectly through gestational weight gain (GWG) and pre-pregnancy body mass index (BMI), both of which are key factors linked to pregnancy outcomes. Excessive or insufficient GWG is associated with negative maternal and fetal consequences, though evidence on the benefits of optimal GWG remains limited [10].

Despite these findings, several gaps persist in the literature. Many studies are cross-sectional, limiting the ability to establish causality between macronutrient intake and GDM risk. Additionally, inconsistent definitions and variations in dietary reference guidelines across studies complicate comparisons of findings. There is also an insufficient focus on the role of pre-pregnancy nutrition in influencing GDM risk. Furthermore, people around the globe follow different dietary patterns, such as Western, Mediterranean, or Asian diets, which may affect the applicability of study results across different populations. Moreover, the interaction between different macronutrients and their collective impact on GDM risk is not well understood, and much of the existing research is concentrated on Western populations, with limited studies addressing diverse dietary patterns in other regions. To address these gaps, this study aimed to evaluate the impact of both pre-pregnancy and during-pregnancy, until mid-gestation, macronutrient intake on GDM risk in a well-defined cohort and also assessed dietary shifts from pre-pregnancy to pregnancy to provide clearer insights on optimal macronutrient distributions in maternal diets to reduce GDM risk and improve pregnancy outcomes.

## 2. Materials and Methods

### 2.1. Study Design and Population

The current study used data from the pregnant cohort BORN2020, an ongoing Greek prospective epidemiologic study, run by the 3rd Department of Obstetrics and Gynecology, School of Medicine, Faculty of Health Sciences, Aristotle University of Thessaloniki, Greece. Participants were recruited at their first planned perinatal visit for their routine pregnancy scan. Two researchers (AT and AA) interviewed the participants.

The study design was approved by the Bioethics Committee of Aristotle University of Thessaloniki, Greece (5/12 April 2022). After obtaining a written informed consent, two planned interviews followed to collect all data.

### 2.2. Diagnosis of GDM

The diagnosis of GDM was based on the criteria established by the Hellenic Society of Obstetricians and Gynecologists (HSOG), which were derived from the Hyperglycemia and Adverse Pregnancy Outcomes (HAPO) study [11]. These criteria include meeting one or more of the following thresholds: a fasting blood glucose level of ≥92 mg/dL, a 1 h post-oral glucose tolerance test (OGTT) glucose level of ≥180 mg/dL, or a 2 h post-OGTT glucose level of ≥153 mg/dL.

### 2.3. Dietary Assessment

The recorded intake data were entered into Nutrisurvey (Windows, v2007) for analysis, capturing macronutrient intake across two defined periods: period A, covering the six months prior to pregnancy, and period B, spanning from conception to mid-gestation at 24 weeks. Dietary data were collected at specific time points: the first assessment was conducted at the 11^+0^–13^+6^ week perinatal visit for pre-pregnancy intake, using a validated food frequency questionnaire (FFQ) [12]. The second assessment at 21^+0^–23^+6^ weeks captured dietary intake during pregnancy until mid-gestation. Each interview lasted approximately 20 min. In our study, we focused on assessing key macronutrients, including total energy, carbohydrates, proteins, and fats. Trans fatty acids (TFAs) were excluded due to regulatory measures in Greece aimed at minimizing their presence in the population’s diet.

### 2.4. Statistical Methods

Statistical analyses were conducted to characterize the variables and assess group differences. For continuous variables, normality was assessed using the Kolmogorov–Smirnov test. Normally distributed variables were summarized using means and standard deviations (SDs), whereas non-normally distributed variables were described with medians and interquartile ranges (IQRs). Group comparisons were performed using t-tests for normally distributed variables and Mann–Whitney U tests for non-normally distributed variables. Categorical variables were summarized as counts and percentages, with group comparisons conducted using chi-squared tests. For binary outcomes with small sample sizes, Fisher’s exact test was employed. The R programing language (v4.2.1) was used for the statistical analysis and implementation.

To assess the relationship between macronutrient intake and GDM, a multinomial logistic regression model was applied to calculate adjusted odds ratios (aORs) with 95% confidence intervals (CIs). Covariates included maternal age (continuous variable, measured in years), parity (binary variable, 0 or ≥1), physical activity level (number of times walking more than 10 min per day), smoking (binary variable, current smoker or non-smoker), thyroid status (e.g., hypothyroidism, Hashimoto’s thyroiditis, and hyperthyroidism; assessed as binary variable, normal or abnormal thyroid function), use of assisted reproductive methods (ART; binary variable, yes/no), energy intake (continuous variable, measured in kcal/day), BMI (continuous variable, measured in kg/m^2^), supplementation intake (binary variable, yes/no), and gestational weight gain until 24 weeks of gestation ( continuous variable, measured as the difference in weight, in kilograms, between weeks 11 and 24 of gestation). For the purposes of this analysis, we hypothesized that the weight at week 11 is approximately equivalent to the pre-pregnancy weight, allowing gestational weight gain to be calculated starting from this point. To evaluate adherence to European Food Safety Authority (EFSA) guidelines, participants’ macronutrient intakes were classified as a binary variable indicating whether intakes were above or below the recommended levels. Associations between exceeding EFSA recommendations and GDM risk were assessed using multinomial logistic regression, with adjustments for the same covariates as in the macronutrient analysis. Differences in macronutrient intake between the pre-pregnancy period (period A) and the pregnancy period (period B) were analyzed using multinomial logistic regression with the same set of covariates. Nutrients analyzed included energy, protein, fat, carbohydrates, dietary fiber, and other relevant macronutrients.

## 3. Results

GDM individuals had a significantly higher mean maternal age compared to non-GDM women (34.15 ± 4.48 years vs. 32.1 ± 4.89 years, *p* < 0.0001), with a larger proportion being > 35 years (43.59% vs. 27.35%, *p* < 0.001). Pre-pregnancy weight and BMI were also significantly elevated among women with GDM. The median pre-pregnancy weight was 65 kg (IQR 59–79) compared to 63 kg (IQR 57–73) in non-GDM women (*p* = 0.008), and the median pre-pregnancy BMI was 23.7 (IQR 21.7–28.5) versus 22.7 (IQR 20.8–26.02, *p* = 0.002). Additionally, a higher percentage of women with GDM had a pre-pregnancy BMI > 30 (21.37% vs. 11.03%, *p* = 0.003). Smoking was more common in the GDM group (17.95% vs. 8.82%, *p* = 0.004). No significant differences were found in the remaining maternal characteristics between the two groups (Table 1).

Analysis of macronutrient intake and its association with GDM risk as seen in Table 2 indicates that dietary fiber intake during pregnancy until mid-gestation was positively associated with GDM risk (aOR = 1.05 [1.00, 1.10], *p* = 0.045), though no association was observed pre-pregnancy. Total protein intake during pregnancy until mid-gestation was also significantly associated with GDM risk (aOR = 1.02 [1.00, 1.04], *p* = 0.042), as was protein expressed as a percentage of energy intake (aOR = 1.08 [1.01, 1.17], *p* = 0.023). No significant associations were found for energy intake, total carbohydrate, total fat, or specific types of fats (e.g., saturated fats, monounsaturated fats, and polyunsaturated fats) with GDM in any time-period.

As shown in Table 3, pre-pregnancy energy intake was higher than the EFSA recommendation in the GDM group (aOR = 1.99 [1.37, 2.86], *p* = 0.001) in comparison to the non-GDM group. During mid-gestation, energy intake above recommendations showed no significant association with GDM risk (aOR = 1.24 [0.68, 2.18], *p* = 0.46). For dietary fiber, no significant associations with GDM risk were observed either pre-pregnancy (aOR = 1.65 [0.47, 5.07], *p* = 0.40) or during mid-gestation (aOR = 1.24 [0.40, 3.41], *p* = 0.69).

Total carbohydrate intake also showed no significant associations with GDM risk in either period (pre-pregnancy: aOR = 1.96 [0.52, 6.24], *p* = 0.28; mid-gestation: aOR = 1.05 × 10^−7^ [1.22 × 10^−60^, 9.82 × 10^9^], *p* = 0.98). Similarly, total fat intake was not significantly associated with GDM risk in either period (pre-pregnancy: aOR = 0.84 [0.55, 1.32], *p* = 0.46; mid-gestation: aOR = 1.01 [0.62, 1.72], *p* = 0.96). Protein intake, whether pre-pregnancy or during mid-gestation, also showed no significant associations with GDM risk (pre-pregnancy: aOR = 1.28 [0.89, 1.84], *p* = 0.17; mid-gestation: insufficient data for analysis). Finally, data for eicosapentaenoic acid (EPA) and docosahexaenoic acid (DHA) intake were insufficient to allow for conclusions in either period.

The comparison of maternal macronutrient intake between period B (during pregnancy until mid-gestation) and period A (pre-pregnancy) highlighted significant changes in dietary patterns (Table 4). Protein intake increased significantly during pregnancy, as reflected by a higher absolute protein intake (aOR = 1.00 [1.00, 1.01], *p* = 0.007), but not in protein as a percentage of energy intake (aOR = 1.02 [0.98, 1.07], *p* = 0.27). Total fat intake also showed a significant increase during pregnancy (aOR = 1.00 [1.00, 1.01], *p* = 0.022), though the percentage of energy from fat remained unchanged (aOR = 1.01 [0.99, 1.02], *p* = 0.21).

The most pronounced change was observed in dietary fiber intake, which increased significantly from pre-pregnancy to pregnancy (aOR = 1.07 [1.04, 1.10], *p* < 0.0001). This suggests a notable dietary shift to include more fiber-rich foods during pregnancy until mid-gestation. Similarly, vegetable protein and animal protein intakes both increased significantly during pregnancy until mid-gestation, with an increase in vegetable protein (aOR = 1.01 [0.99, 1.03], *p* = 0.044) and animal protein (aOR = 1.00 [1.00, 1.01], *p* = 0.019).

In terms of specific fatty acids, a significant increase in monounsaturated fatty acid (MUFA) intake was observed (aOR = 1.01 [1.00, 1.02], *p* < 0.0001), while no significant changes were detected for saturated fatty acids (SFAs), polyunsaturated fatty acids (PUFAs), or omega-3 fatty acids such as EPA and DHA. Carbohydrate intake, both in absolute terms and as a percentage of energy, did not differ significantly between the two periods (*p* = 0.39 for absolute intake, and *p* = 0.15 for percentage of energy).

Supplementary Table S1 includes the reference macronutrient guidelines for non-pregnant and pregnant women provided by the EFSA.

## 4. Discussion

The current study assessed maternal macronutrient intake in the pre-pregnancy and during the first half of pregnancy periods. The findings indicated that (i) women with GDM were of higher age and more likely to be > 35 years compared to the non-GDM women, (ii) pre-pregnancy weight, BMI, and BMI > 30 were higher in GDM, (iii) smoking was more common in GDM, (iv) higher pre-pregnancy energy intake (exceeding EFSA recommendations) was associated with an increased risk of GDM, (v) higher dietary fiber and higher protein intake during pregnancy until mid-gestation were associated with increased GDM risk, (vi) increases in dietary fiber, protein, fat, vegetable protein, animal protein, and MUFA intake from pre-pregnancy to pregnancy were associated with higher GDM risk.

Maternal age is an established risk factor for GDM, with the risk increasing linearly with advancing maternal age, as evidenced by a recent meta-analysis of 24 studies [13]. For women aged ≥ 35 years, the odds of developing GDM increased significantly, with a 2.73-fold higher risk for those aged 35–39 years and a 3.54-fold higher risk for those aged ≥ 40 years compared to the reference group aged 25–29 years. Additionally, the dose–response analysis revealed that for each year beyond 18 years of age, the risk of GDM increased by 7.90%, emphasizing the cumulative effect of maternal age on GDM risk. In our study, we observed a similar pattern, with women aged > 35 years showing a higher prevalence of GDM compared to younger age groups, aligning with the established evidence of maternal age as a significant contributor to GDM risk.

In our population, women with GDM had higher weight and BMI pre-pregnancy, with 21% of GDM cases classified as obese (BMI > 30). Additionally, women that developed GDM more often reported pre-pregnancy energy intakes which exceeded the EFSA. This finding is consistent with the role of pre-pregnancy weight and BMI as significant risk factors for GDM, as excessive energy intake contributes to weight gain and elevated BMI prior to pregnancy [14]. Similarly, Lewandowska et al. demonstrated that pre-pregnancy obesity significantly increases GDM risk (aOR 2.99–11.88), even after accounting for gestational weight gain, stressing the importance of preconception dietary and lifestyle interventions [15].

Smoking exposure was associated with increased risk for GDM. Data on smoking status before pregnancy have been associated with an increased risk of GDM, likely through metabolic and hormonal disruptions associated with long-term smoking [16]. However, there are mixed findings in the literature regarding smoking during pregnancy and GDM risk, which often show no significant association, and may reflect differences in smoking intensity, timing, or cessation heterogeneity in study designs, populations, and diagnostic criteria for GDM [17]. The results of the current study suggest a correlation between smoking and GDM, potentially driven by the contribution of pre-pregnancy smoking, which has been consistently associated with higher odds of GDM.

The role of dietary fiber in GDM risk is complex and appears to depend on various factors, including the source and timing of fiber intake. While high total fiber and fruit fiber intake have been shown to have protective effects against GDM, particularly during the second trimester, other studies have reported that specific types of fiber, such as cereal fiber, might be associated with an increased GDM risk [18]. This may reflect broader dietary patterns, such as the consumption of refined grains or added sugars that often accompany cereal fiber, which could counteract its potential benefits. Fruits are widely perceived as a healthy dietary choice, and pregnant women might increase their fruit intake due to this perception. However, the sugar content in fruits varies significantly by type, with some fruits containing higher natural sugar levels than others. This could potentially influence glucose metabolism and, in turn, GDM risk, especially in susceptible individuals. In our analysis, we observed an association between increased dietary fiber intake during pregnancy until mid-gestation and a higher risk of GDM, along with an increase in fiber intake from pre-pregnancy to pregnancy correlating with higher odds of GDM. Although the current study did not differentiate between types of fiber, these results possibly suggest that dietary changes during pregnancy until mid-gestation, potentially driven by efforts to adopt healthier eating habits, might introduce metabolic shifts that influence glucose regulation.

Protein intake and its sources appear to play a significant role in GDM risk during pregnancy. In our analysis, higher absolute protein intake and an increased percentage of total energy derived from protein during pregnancy until mid-gestation were associated with elevated odds of developing GDM, a finding which is in line with available evidence [19]. Furthermore, increases in protein intake from pre-pregnancy to pregnancy, from either animal or vegetable sources, were similarly linked to higher GDM risk, suggesting that total protein intake, rather than the source, may be the culprit. This may be due, in part, to the accompanying intake of saturated fats or specific amino acids present in high-protein foods that influence insulin sensitivity and glucose metabolism. Results from a recent meta-analysis by Talebi et al. showed a positive linear relationship between total protein, animal protein, and red and processed meat consumption, with GDM risk rising with increased intake (RR 1.32 per 5% increment in animal protein; 1.94 per 100 g/day increment in red meat) [20]. These results corroborate our findings, reinforcing the notion that total protein intake, irrespective of source, may contribute to GDM development.

Data on dietary shifts from pre-pregnancy to pregnancy are limited. To our knowledge, this is the first study conducted in the Greek population that assesses this effect. Specifically, in our analysis, we observed an increase in total fat intake from pre-pregnancy to pregnancy that was associated with a higher risk of GDM. Results from recent data report that higher dietary fat intake during pregnancy is linked to an increased risk of GDM, possibly by affecting glucose regulation [21]. Furthermore, our findings regarding the association between an increased shift in MUFA intake and GDM risk underscore the significant role of dietary fats in modulating this condition. This change from the pre-pregnancy to the first half of pregnancy period reflects the influence of changes in fat quality and overall energy balance on GDM risk. Notably, if the increased MUFA intake stems from processed or high-calorie foods, rather than healthy sources like olive oil, it may contribute to excess energy intake and weight gain, thereby exacerbating the risk of GDM [22].

### 4.1. Strengths and Limitatons

This study assessed both pregnancy and pre-pregnancy, capturing dietary habits prior to disease onset and enhancing the ability to infer causality between macronutrient intake and GDM risk. Additionally, this study’s substantial sample size of 797 pregnant women, including 117 diagnosed with GDM, provided adequate statistical power to detect significant associations. Assessing dietary intake at two distinct time points allowed us to evaluate changes in macronutrient consumption, offering novel insights into how these shifts relate to GDM risk.

As with any study, this research has limitations that should be considered when interpreting the findings. Some observed effect sizes were small, and the use of self-reported dietary data introduces potential recall bias, though interviews by experienced health care professionals helped mitigate this issue. This study’s focus on a specific Greek cohort provides valuable insights but may limit generalizability to other populations. Additionally, data collection occurred during the lockdown, which may have influenced participants’ dietary habits, lifestyle choices, or healthcare access. However, this was partially accounted for by addressing macronutrient intakes both before and during gestation, thereby comparing participants under similar conditions. Lastly, this study did not differentiate between macronutrient sources, highlighting an area for future research. Despite these limitations, this study makes a significant contribution to understanding the association between maternal macronutrient intake and GDM risk, particularly during pre-pregnancy and mid-gestation.

### 4.2. Clinical Implications

The findings of the current study highlight the importance of preconception nutritional counseling as a potential strategy to reduce the risk of GDM [23] and to maintain the glycemic response [24]. Specifically, the significant association between pre-pregnancy energy intake exceeding EFSA recommendations and increased GDM risk suggests that interventions targeting dietary patterns prior to conception could play a critical role in mitigating GDM incidence. It is also essential to maintain a balanced macronutrient intake throughout the gestation period and to avoid an emphasis on and overconsumption of specific macronutrients, as certain components of high-protein diets, such as saturated fats or particular amino acids, may adversely affect glucose metabolism and insulin sensitivity [20].

Furthermore, the observed dietary shifts during pregnancy highlight the need for continuous dietary monitoring of specific dietary patterns, and not only macronutrient intakes [25], and tailored guidance for high-risk individuals, particularly those with elevated energy or specific macronutrient intakes.

## 5. Conclusions

Monitoring dietary patterns both before and during pregnancy until mid-gestation, focusing on pre-pregnancy energy overload and particularly focusing on shifts in macronutrient intake, might be important. The observed association between increased shifts in fat and protein consumption, especially shifts in MUFA intake from pre-pregnancy to pregnancy, and higher GDM risk highlights the need to address not only the quantity but also the quality of dietary fats. Consumption of healthy fat sources should be emphasized for both periods, while also minimizing processed or high-calorie foods that may contribute to excessive weight gain and impaired glucose regulation. Larger and more diverse studies are necessary to confirm these associations to refine nutritional recommendations for women at risk of GDM.

## Figures and Tables

**Table 1:** Maternal characteristics of GDM and non-GDM women.

Maternal Variables	GDM (Ν = 117)	Non-GDM (Ν = 680)	*p* Value	Comparison
MA (mean, SD)	34.15 (±4.48)	32.1 (±4.89)	*p* < 0.0001 ***	↑ in GDM
MA > 35 (n %)	51 (43.59%)	186 (27.35%)	*p* < 0.001 ***	↑ in GDM
Weight before pregnancy (median (IQR))	65 (59, 79)	63 (57, 73)	0.008 **	↑ in GDM
Height (cm) (median (IQR))	165 (161, 170)	165 (162, 170)	0.27	-
BMI before pregnancy (median (IQR))	23.7 (21.7, 28.5)	22.7 (20.8, 26.02)	0.002 **	↑ in GDM
BMI > 25 before pregnancy (median (IQR))	46 (39.32%)	214 (31.47%)	0.12	-
BMI > 30 before pregnancy (median (IQR))	25 (21.37%)	75 (11.03%)	0.003 **	↑ in GDM
Smoking	21 (17.95%)	60 (8.82%)	0.004 **	↑ in GDM
Thyroid disease	13 (11.11%)	93 (13.68%)	0.54	-
Parity 0 1 2 3 4	60 (51.28%) 44 (37.61%) 12 (10.26%) 1 (0.855%) 0 (0%)	347 (51.03%) 253 (37.21%) 69 (10.15%) 9 (1.32%) 2 (0.294%)	0.96 0.98 0.9 0.98 -	- - - - -
Planned pregnancy (n%)	103 (88.03%)	582 (85.71%)	0.6	-
Conception with ART (n%)	11 (9.4%)	48 (7.06%)	0.48	-

IQR: interquartile range, represented by the 25th percentile, median, and 75th percentile; n: number of participants; SD: standard deviation; MA: maternal age in years; BMI: body mass index calculated as weight in kilograms divided by height in meters squared (kg/m^2^). Thyroid disease includes conditions such as hypothyroidism, hyperthyroidism, and Hashimoto’s disease. Conception ART refers to conception through assisted reproductive technologies; the “↑” symbol indicates that values in the GDM group are higher than in the non-GDM group; “**” denotes *p* < 0.01, and “***” *p* < 0.001. Descriptive statistics include mean (SD), percentiles, or proportions. *p*-values were derived using the *t*-test for normally distributed continuous variables, the Mann–Whitney U test for non-normally distributed variables, and the chi-squared test for categorical variables.

**Table 2:** Macronutrient intake and GDM risk among GDM (N = 117) and non-GDM (N = 680) women.

	Period A			Period B		
Macronutrients	Reference Values	*p*-Value (aOR)	aOR (95% CI)	Reference Values	*p*-Value (aOR)	aOR (95% CI)
Energy (E)	AR 2147 kcal/day or 2072 kcal/day depending on age	0.53	1 (0.99, 1)	AR (+) 70 + 260 kcal/day (1st and 2nd trimester)	0.63	1 (0.99, 1)
Carbohydrates absolute value		0.25	0.99 (0.99, 1)		0.44	0.99 (0.99, 1)
-Dietary fiber	AI 25 g/day	0.89	1 (0.94, 1.05)	AI 25 g/day	0.045 *	1.05 (1, 1.1)
-Total carbohydrates %	45–60 E%	0.23	0.98 (0.96, 1)	45–60 E%	0.25	0.98 (0.96, 1)
Fats absolute value		0.24	1 (0.99, 1.02)		0.88	1 (0.98, 1.01)
-Eicosapentaenoic acid and docosahexaenoic acid (EPA and DHA)	250 mg/day DHA + EPA	0.69	1.47 (0.21, 9.48)	250 (+) 100-200 mg/day DHA + EPA	0.44	1.97 (0.33, 10.37)
-Saturated fatty acids (SFAs)	AI ALAP	0.47	1.01 (0.97, 1.04)	AI ALAP	0.57	0.99 (0.95, 1.02)
-Total fat %	RI 20–35%	0.25	1.01 (0.99, 1.03)	RI 20–35%	0.71	1 (0.98, 1.02)
Protein absolute value	AR 0.66 g/kg bw per day	0.56	0.99 (0.97, 1.01)	AR (+) 0.52 + 7.2 g/kg bw per day (1st and 2nd trimester)	0.042 *	1.02 (1, 1.04)
-Protein %	-	0.74	0.98 (0.91, 1.06)		0.023 *	1.08 (1.01, 1.17)
-Vegetarian protein	-	0.49	0.98 (0.94, 1.02)		0.38	1.02 (0.97, 1.06)
-Animal protein	-	0.74	0.99 (0.98,1.01)		0.11	1.01 (0.99, 1.03)
Monounsaturated fatty acids (MUFAs)	-	0.34	1 (0.99, 1.02)		0.64	1 (0.98, 1.02)
Polyunsaturated fatty acids (PUFAs)	-	0.53	1.01 (0.96, 1.05)		0.85	1 (0.94, 1.06)

AR: average requirement; AI: adequate intake; E%: percentage of energy; ALAP: as low as possible; RI: reference intake; MUFAs: monounsaturated fatty acids; PUFAs: polyunsaturated fatty acids; EPA and DHA: eicosapentaenoic acid and docosahexaenoic acid; “-” means there were not enough data to draw conclusions; “*” denotes *p* < 0.05. *p*-values and adjusted odds ratios (aORs) were calculated using multinomial logistic regression models, adjusting for maternal age, parity, physical activity, smoking, thyroid status, ART use, energy intake, BMI, supplementation intake, and gestational weight gain. All reference values were sourced from the EFSA Dietary Reference Values Database (https://multimedia.efsa.europa.eu/drvs/index.htm, access date: 28 December 2024).

**Table 3:** Macronutrient intake among GDM (N = 117) and non-GDM (N = 680) women, in comparison to EFSA guidelines.

	Period A			Period B		
Macronutrients	Above EFSA Guidelines	*p*-Value (aOR)	aOR (95% CI)	Above EFSA Guidelines	*p*-Value (aOR)	aOR (95% CI)
Energy (E)	>AR 2147 kcal/day or 2072 kcal/day depending on age	*p* < 0.001 ***	1.99 (1.37, 2.86)	>AR (+) 70 + 260 kcal/day (1st and 2nd trimester)	0.46	1.24 (0.68, 2.18)
-Dietary fiber	>AI 25 g/day	0.4	1.65 (0.47, 5.07)	>AI 25 g/day	0.69	1.24 (0.4, 3.41)
-Total carbohydrates %	>45–60 E%	0.28	1.96 (0.52, 6.24)	>45–60 E%	0.98	1.05 × 10^−7^ (1.22 × 10^−60^, 9.82 × 10^9^)
Fats						
-Eicosapentaenoic acid and Docosahexaenoic acid (EPA and DHA)	>250 mg/day DHA + EPA	-	- (-,-)	>250 (+) 100-200 mg/day DHA + EPA	-	- (-,-)
-Total fat %	>RI 20–35%	0.46	0.84 (0.55, 1.32)	>RI 20–35%	0.96	1.01 (0.62, 1.72)
Protein	>AR 0.66 g/kg bw per day	0.17	1.28 (0.89, 1.84)	>AR (+) 0.52 + 7.2 g/kg bw per day (1st and 2nd trimester)	-	- (-,-)

AR: average requirement; AI: adequate intake; E%: percentage of energy; RI: reference intake; EPA and DHA: eicosapentaenoic acid and docosahexaenoic acid; “-” means there were not enough data to draw conclusions; “***” denotes *p* < 0.001. *p*-values and aORs were derived from multinomial logistic regression models, adjusted for the same covariates as in Table 2. All reference values were sourced from the EFSA Dietary Reference Values Database (https://multimedia.efsa.europa.eu/drvs/index.htm, access date 28 December 2024).

**Table 4:** Differences in maternal dietary macronutrient shifts between GDM (N = 117) and non-GDM (N = 680) women from period B (during pregnancy until mid-gestation) in comparison to period A (pre-pregnancy).

Dietary Shifts in Macronutrient Intake Between Pre-Pregnancy (A) and Pregnancy (B)	*p*-Value (aOR)	aOR (95% CI)
Energy (B-A)	0.39	1 (0.99, 1)
Protein (B-A)	0.007 **	1 (1, 1.01)
Protein % (B-A)	0.27	1.02 (0.98, 1.07)
Fat (B-A)	0.022 *	1 (1, 1.01)
Fat % (B-A)	0.21	1.01 (0.99, 1.02)
Carbohydrates (B-A)	0.39	1 (0.99, 1)
Carbohydrates % (B-A)	0.15	0.98 (0.97, 1)
Dietary fiber (B-A)	*p* < 0.0001 ***	1.07 (1.04, 1.1)
Vegetable protein (B-A)	0.044 *	1.01 (0.99, 1.03)
Animal protein (B-A)	0.019 *	1 (1, 1.01)
SFAs (B-A)	0.63	0.99 (0.98, 1)
MUFAs (B-A)	*p* < 0.0001 ***	1.01 (1, 1.02)
PUFAs (B-A)	0.15	1.01 (0.99, 1.04)
EPA and DHA (B-A)	0.2	1.74 (0.72, 3.93)

SFAs: saturated fatty acids; MUFAs: monounsaturated fatty acids; PUFAs: polyunsaturated fatty acids; EPA and DHA: eicosapentaenoic acid and docosahexaenoic acid. *p*-values and aORs were derived from multinomial logistic regression models, adjusted for the same covariates as in Table 2. “*” denotes *p* < 0.05, “**” denotes *p* < 0.01, and “***” denotes *p* < 0.001, indicating the levels of statistical significance.

## Data Availability

Data not available due to institutional restrictions.

## Supplementary Materials

The following supporting information can be downloaded at https://www.mdpi.com/article/10.3390/biomedicines13010057/s1: Table S1. Reference macronutrient guidelines for non-pregnant and pregnant women.

## References

[1] Costanza J., Camanni M., Ferrari M.M., De Cosmi V., Tabano S., Fontana L., Radaelli T., Privitera G., Alberico D., Colapietro P. (2022). Assessment of pregnancy dietary intake and association with maternal and neonatal outcomes. Pediatr. Res..

[2] Sharma S.S., Greenwood D.C., Simpson N.A.B., Cade J.E. (2018). Is dietary macronutrient composition during pregnancy associated with offspring birth weight? An observational study. Br. J. Nutr..

[3] Chen L.-W., Tint M.-T., Fortier M.V., Aris I.M., Bernard J.Y., Colega M., Gluckman P.D., Saw S.-M., Chong Y.-S., Yap F. (2016). Maternal macronutrient intake during pregnancy is associated with neonatal abdominal adiposity: The Growing Up in Singapore Towards healthy Outcomes (GUSTO) Study. J. Nutr..

[4] Soma-Pillay P., Nelson-Piercy C., Tolppanen H., Mebazaa A. (2016). Physiological changes in pregnancy: Review articles. Cardiovasc. J. Afr..

[5] Saldana T.M., Siega-Riz A.M., Adair L.S. (2004). Effect of macronutrient intake on the development of glucose intolerance during pregnancy. Am. J. Clin. Nutr..

[6] Moses R.G., Shand J.L., Tapsell L.C. (1997). The recurrence of gestational diabetes: Could dietary differences in fat intake be an explanation?. Diabetes Care.

[7] Mousa A., Naqash A., Lim S. (2019). Macronutrient and micronutrient intake during pregnancy: An overview of recent evidence. Nutrients.

[8] Thayer Z.M., Rutherford J., Kuzawa C.W. (2020). The maternal nutritional buffering model: An evolutionary framework for pregnancy nutritional intervention. Evol. Med. Public Health.

[9] Bao W., Tobias D.K., Olsen S.F., Zhang C. (2013). Prepregnancy dietary protein intake, major dietary protein sources, and the risk of gestational diabetes mellitus: A prospective cohort study. Diabetes Care.

[10] Goldstein R.F., Abell S.K., Ranasinha S., Misso M.L., Boyle J.A., Harrison C.L., Black M.H., Li N., Hu G., Corrado F. (2018). Gestational weight gain across continents and ethnicity: Systematic review and meta-analysis of maternal and infant outcomes in more than one million women. BMC Med..

[11] Coustan D.R., Lowe L.P., Metzger B.E., Dyer A.R. (2010). The Hyperglycemia and Adverse Pregnancy Outcome (HAPO) study: Paving the way for new diagnostic criteria for gestational diabetes mellitus. Am. J. Obstet. Gynecol..

[12] Apostolopoulou A., Magriplis E., Tsekitsidi E., Oikonomidou A.C., Papaefstathiou E., Tsakiridis I., Dagklis T., Chourdakis M. (2021). Development and validation of a short culture-specific food frequency questionnaire for Greek pregnant women and their adherence to the Mediterranean diet. Nutrition.

[13] Li Y., Ren X., He L., Li J., Zhang S., Chen W. (2020). Maternal age and the risk of gestational diabetes mellitus: A systematic review and meta-analysis of over 120 million participants. Diabetes Res. Clin. Pract..

[14] Ruhstaller K.E., Bastek J.A., Thomas A., Mcelrath T.F., Parry S.I., Durnwald C.P. (2016). The effect of early excessive weight gain on the development of hypertension in pregnancy. Am. J. Perinatol..

[15] Lewandowska M., Więckowska B., Sajdak S. (2020). Pre-pregnancy obesity, excessive gestational weight gain, and the risk of pregnancy-induced hypertension and gestational diabetes mellitus. J. Clin. Med..

[16] Bar-Zeev Y., Haile Z.T., Chertok I.A. (2020). Association between prenatal smoking and gestational diabetes mellitus. Obstet. Gynecol..

[17] Athanasiadou K.I., Paschou S.A., Papakonstantinou E., Vasileiou V., Kanouta F., Kazakou P., Stefanaki K., Kassi G.N., Psaltopoulou T., Goulis D.G. (2023). Smoking during pregnancy and gestational diabetes mellitus: A systematic review and meta-analysis. Endocrine.

[18] Xu Q., Tao Y., Zhang Y., Zhang X., Xue C., Liu Y. (2021). Dietary fiber intake, dietary glycemic load, and the risk of gestational diabetes mellitus during the second trimester: A nested case-control study. Asia Pac. J. Clin. Nutr..

[19] Pang W.W., Colega M., Cai S., Chan Y.H., Padmapriya N., Chen L.-W., Soh S.-E., Han W.M., Tan K.H., Lee Y.S. (2017). Higher maternal dietary protein intake is associated with a higher risk of gestational diabetes mellitus in a multiethnic Asian cohort. J. Nutr..

[20] Talebi S., Ghoreishy S.M., Ghavami A., Sikaroudi M.K., Nielsen S.M., Talebi A., Mohammadi H. (2024). Dose–response association between animal protein sources and risk of gestational diabetes mellitus: A systematic review and meta-analysis. Nutr. Rev..

[21] Talebi S., Zeraattalab-Motlagh S., Rahimlou M., Sadeghi E., Rashedi M.H., Ghoreishy S.M., Mohammadi H. (2024). Dietary fat intake with risk of gestational diabetes mellitus and preeclampsia: A systematic review and meta-analysis of prospective cohort studies. Nutr. Rev..

[22] Schwingshackl L., Strasser B., Hoffmann G. (2011). Effects of monounsaturated fatty acids on glycaemic control in patients with abnormal glucose metabolism: A systematic review and meta-analysis. Ann. Nutr. Metab..

[23] Valkama A., Koivusalo S., Lindström J., Meinilä J., Kautiainen H., Stach-Lempinen B., Rönö K., Klemetti M., Pöyhönen-Alho M., Tiitinen A. (2016). The effect of dietary counselling on food intakes in pregnant women at risk for gestational diabetes: A secondary analysis of a randomised controlled trial RADIEL. Eur. J. Clin. Nutr..

[24] Valença M.C.T., França M.S., Mattar R., Dualib P.M., Sanchez V.H.S., de Almeida-Pititto B., Júnior E.A., Traina E. (2024). Nutritional guidance through digital media for glycemic control of women with gestational diabetes mellitus: A randomized clinical trial. J. Perinat. Med..

[25] Fagherazzi S., Farias D.R., Belfort G.P., dos Santos K., de Lima T.S.V., dos Santos M.S., Saunders C. (2021). Impact of the Dietary Approaches to Stop Hypertension (DASH) diet on glycaemic control and consumption of processed and ultraprocessed foods in pregnant women with pre-gestational diabetes mellitus: A randomised clinical trial. Br. J. Nutr..

