# Supplementary material for: Maternal Macronutrient Intake and Associated Risk for Gestational Diabetes Mellitus: Results from the BORN2020 Study
Source: Biomedicines. 2024 Dec 29;13(1):57. doi: 10.3390/biomedicines13010057 (PMC11763321; doi:10.3390/biomedicines13010057)
Supplement: Supplementary file 1 [file biomedicines-13-00057-s001.zip › biomedicines-3372709-supplementary.pdf]

Supplementary material

Table S1. Reference macronutrient guidelines for non-pregnant and pregnant women.

Macronutrients	Above EFSA guidelines	Reference values	**p-value (aOR)**	**aOR (95% CI)**
Energy (E)	>AR 2147 kcal/day or 2072 kcal/day depending on age	>AR (+) 70+260 kcal/day (1st and 2nd trimester)	0.46	1.24 (0.68,2.18)
-Dietary fiber	>AI 25 g/day	>AI 25 g/day	0.69	1.24 (0.4,3.41)
-Total carbohydrates%	>45-60 E%	>45-60 E%	0.98	$1.05*10^{-7}$ ($1.22*10^{-60}$,$9.82*10^{9}$)
Fats				
-Eicosapentaenoic acid, Docosahexaenoic acid (EPA, DHA)	>250 mg/day DHA + EPA	>250 (+) 100-200 mg/day DHA + EPA	-	- (-,-)
-Total fat %	>RI 20-35%	>RI 20-35%	0.96	1.01 (0.62,1.72)
Protein	>AR 0.66 g/kg bw per day	>AR (+) 0.52 + 7.2 g/kg bw per day (1st and 2nd trimester)	-	- (-,-)

Source: https://multimedia.efsa.europa.eu/drvs/index.htm, access date 28/12/2024
